# Effects of Sitagliptin and Celery Seed Extract on Corneal Nerve Morphology and Sensory Dysfunction in Diabetic Rats

**DOI:** 10.3390/nu18142243

**Published:** 2026-07-09

**Authors:** Samea Khan, Maria Markoulli, Lamia Nureen, Nick Di Girolamo, Mark Willcox

**Affiliations:** 1 School of Optometry and Vision Science, University of New South Wales, Sydney, NSW 2052, Australia; m.markoulli@unsw.edu.au; 2 School of Biomedical Sciences, University of New South Wales, Sydney, NSW 2052, Australia; lamianu13@gmail.com (L.N.); n.digirolamo@unsw.edu.au (N.D.G.)

**Keywords:** type 2 diabetes, sitagliptin, celery seed extract, corneal nerve fibres, sensory responses

## Abstract

**Background:** Diabetic peripheral neuropathy (DPN) is a common complication of diabetes, characterised by sensory dysfunction and progressive loss of small nerve fibres. Corneal nerves are among the earliest fibres affected and may serve as sensitive markers for early detection and therapeutic intervention. Sitagliptin and celery seed extract have demonstrated anti-hyperglycaemic and neuroprotective properties in experimental diabetes; however, their effects on corneal nerve morphology and function remain unknown. **Purpose**: The present study aimed to investigate the effects of sitagliptin and celery seed extract on corneal nerve morphology, corneal sensitivity, and sensory behaviour in a high-fat diet, streptozotocin-induced rat model of type 2 diabetes. **Methods:** Male Sprague Dawley rats (*n* = 24) were used to induce type 2 diabetes by combining a high-fat diet with streptozotocin. Diabetic rats were treated with sitagliptin (30 mg/kg per day for 4 weeks) or celery seed extract (100 mg/kg per day for 4 weeks). Fasting blood glucose levels were measured throughout the monitoring period. Corneal nerve fibre parameters, including corneal nerve fibre length (CNFL), density (CNFD), and tortuosity, were assessed in βIII-tubulin-stained whole-mount corneas. Corneal sensitivity was measured with the Cochet–Bonnet esthesiometer. Sensory behaviour was evaluated using hot- and cold-water tail immersion and an acetone drop test. **Results:** Diabetes significantly reduced the CNFL (39.8 ± 4.7 vs. normal: 71.7 ± 7.3 mm/mm^2^; *p* < 0.05) and CNFD (7.1 ± 0.9 vs. normal: 10.6 ± 1.2%; *p* < 0.05) and increased corneal nerve tortuosity (5.8 ± 0.2 vs. normal: 5.1 ± 0.2; *p* < 0.05). Both sitagliptin and celery seed extract significantly increased the CNFL (68.8 ± 4.5 and 65.8 ± 6.5 mm/mm^2^, respectively) compared to the untreated diabetic group. Tortuosity was significantly decreased in both the sitagliptin (4.3 ± 0.1) and celery seed extract (4.9 ± 0.1) groups compared with the untreated diabetic group. However, the CNFD showed only a modest increase after either treatment. A significant decrease in corneal sensitivity was also observed in rats following diabetes induction (5.8 ± 0.1 in normal vs. 4.5 ± 0.1 cm in diabetic rats; *p* < 0.05). This was rescued with sitagliptin and celery seed extract treatment (5.5 ± 0.1 and 5.3 ± 0.1 cm, respectively; *p* < 0.05). Compared with the non-diabetic controls, diabetic rats showed significantly shorter withdrawal latencies in both the hot- (3.9 ± 0.3 vs. 7.6 ± 1.1 s) and cold-water tests (4.3 ± 0.5 vs. 8.1 ± 0.9 s), indicating thermal sensitivity. By contrast, diabetic rats showed a significantly longer response time in the acetone drop test (8.0 ± 0.7 vs. 3.6 ± 0.4 s), indicating altered cold sensitivity (*p* < 0.05) for all responses. Treatment with sitagliptin and celery seed extract significantly reversed these changes, as evidenced by increased withdrawal latency in the cold-water test and decreased response time in the acetone drop test. However, this improvement was not significant in the hot-water test. **Conclusions:** Sitagliptin and celery seed extract restored corneal nerve architecture, corneal sensitivity, and sensory dysfunction in diabetic rats, suggesting the therapeutic potential for DPN.

## 1. Introduction

Diabetes is a major global public health challenge, affecting around 589 million adults worldwide [1]. It is associated with many complications, including diabetic peripheral neuropathy (DPN), which is common and debilitating [2]. DPN affects both large and small nerve fibres, causing pain, sensory loss, impaired thermal perception, and reduced quality of life [3]. Previous research has shown that small nerve fibre dysfunction occurs before structural nerve damage, making it an early marker for detection and treatment [4]. Experimental and clinical studies have indicated that DPN is caused by persistent hyperglycaemia, insulin resistance, oxidative stress, inflammation, mitochondrial dysfunction, and microvascular damage [5]. Hyperglycaemia contributes to neuronal damage through oxidative stress and apoptosis [6,7]. DPN affects approximately 50% of people with type 2 diabetes and involves the gradual loss of peripheral nerves from distal to proximal regions. Patients with DPN may experience various symptoms, including spontaneous pain, allodynia, and hyperalgesia, in approximately 10–20% of cases [8]. Currently, there is no known cure for DPN, and prevention and symptom management remain essential priorities [9].

Current treatment options for DPN are very limited and focus on neuropathic pain management instead of disease modification. Although pharmacological options, such as pregabalin, duloxetine, and capsaicin, are being investigated, the effectiveness of treatment remains limited, and many patients experience inadequate symptom relief [10,11].

The common diagnosis of DPN involves evaluating clinical symptoms, performing a neurological examination, and conducting quantitative sensory testing and nerve conduction studies, which primarily assess large-fibre dysfunction and may not detect early small-fibre damage [12,13]. Consequently, much attention has been drawn towards the cornea, the most densely innervated tissue of the human body, which has emerged as a sensitive site for assessing small fibre neuropathy [14]. The subbasal corneal nerve plexus is primarily composed of small sensory fibres, which are susceptible to metabolic injury [15].

Rodent models are widely used to study DPN, and the high-fat diet (HFD) combined with low-dose streptozotocin (STZ) closely replicates key features of type 2 diabetes [16]. This model integrates insulin resistance with partial β-cell dysfunction, leading to hyperglycaemia, altered body composition, sensory abnormalities, and peripheral nerve injury [17,18]. A reduction in the corneal nerve fibre length and density has been reported in both patients with diabetes and animal models of diabetic neuropathy [19,20]. In addition to structural changes, corneal sensitivity provides functional insights into trigeminal nerve integrity, allowing for a comprehensive assessment [14].

Sitagliptin, a dipeptidyl peptidase-4 (DPP-4) inhibitor widely used for glycaemic control and the treatment of type 2 diabetes [18,21], has demonstrated potential neuroprotective effects beyond its glucose-lowering ability [22]. Experimental studies have reported improvements in thermal pain sensitivity, motor function, and nerve structure, along with a reduction in oxidative stress, inflammation, and neuronal apoptosis in a diabetic rat model [22,23,24]. Collectively, these findings suggest that sitagliptin may have therapeutic potential for attenuating diabetic neuropathy. However, its effects on corneal nerve changes and ocular sensory function remain unknown.

Plant-derived therapies are increasingly explored in preclinical and clinical studies due to their ability to target multiple pathological pathways [25,26]. *Apium graveolens* (celery) belongs to the Apiaceae family and is widely cultivated as both a food and medicinal plant. Different parts of the plant, including the leaves, stalks, roots, and particularly the seeds, have been used traditionally for their diuretic, anti-inflammatory, antioxidant, and metabolic effects [27,28]. Celery seeds contain several bioactive compounds, including flavonoids, phenolic acids, and phthalides, with antioxidant, anti-inflammatory, and anti-hyperglycaemic effects [29]. Celery seed extracts significantly lower blood glucose levels and improve lipid profiles [30] and restore antioxidant defence systems [31]. Additionally, celery-derived compounds modulate the pathways involved in oxidative stress, inflammation, and apoptosis, which are fundamental to the pathogenesis of DPN [32]. Celery seeds contain 3-n-butylphthalide (NBP), a bioactive compound with well-established neuroprotective properties, including improved neuronal survival, reduced oxidative damage, and enhanced mitochondrial function [33,34]. The diverse actions of celery seeds and their bioactive constituents suggest strong therapeutic potential for mitigating nerve damage. Despite these promising properties, the effects of celery seed extract on corneal nerve integrity and sensory dysfunction in diabetic corneal neuropathy remain unexplored.

This study aimed to investigate and compare the therapeutic effects of sitagliptin and celery seed extract on corneal nerve structure and sensory dysfunction in an HFD + STZ-induced type 2 diabetes rat model. Confocal microscopy-based nerve analysis and sensory function tests were used as surrogate markers for the development and progression of DPN and to assess the diagnostic and therapeutic potential of the interventions for DPN. We hypothesised that both interventions would ameliorate diabetes-induced neural and sensory deficits.

## 2. Materials and Methods

### 2.1. Ethics Approval

All experiments were conducted in accordance with the protocol (23/106A) approved by the University of New South Wales (UNSW) animal ethics committee. Animals were treated in line with the institutional guidelines for the use of animals in research.

### 2.2. Animals

For this study, *n* = 24 male Sprague Dawley (SD) rats aged 14–16 weeks were sourced from Animal Services–Animal Resources Centre (ARC, Perth, WA, USA) and housed in a certified animal facility at the University of New South Wales, Sydney, Australia. Animals were group-housed, 2 per cage, in a rat colony room with the temperature maintained at 18–22 °C. They were kept in ventilated cages under a 12-h light/dark cycle. Experimental rats had free access to food and water. After arrival, animals were acclimatised for 1 week to the animal house conditions before the experiment began. Sample size estimation was conducted using G*Power 3.1, using a two-tailed independent-samples t-test with α = 0.05 and 95% power. The corneal nerve fibre length (CNFL) estimates were based on a previous study [20], which reported a mean CNFL of 6.2 ± 0.7 mm/mm^2^ in diabetic rats and 8.6 ± 0.9 mm/mm^2^ in the controls. These figures produced an effect size of d = 2.99, indicating a minimum sample size of 5 animals per group. To account for possible attrition during diabetes induction and follow-up, 6 animals were assigned to each group.

### 2.3. Diet

Rats were maintained on ad libitum standard chow (Gordon’s chow, 11 kJ/g, 65% energy as carbohydrate, 22% protein, 13% fat) during the acclimatisation (1–2 weeks) period. After acclimatisation, rats were fed a custom-made HFD (TD:06415), which was designed to resemble the research diet D12451, containing 45% kcal as fat derived from lard [20]. Rats were kept on this diet for the duration of the study (16 weeks), except for the healthy control group, which was fed standard chow throughout.

### 2.4. Chemicals

STZ was purchased from Sigma-Aldrich, supplied by Merck Life Science Pty Ltd., Bayswater, VIC, Australia (Catalogue # S0130), and sitagliptin was sourced from Abcam, Inc., Cambridge, UK (Catalogue # ab142101). Rabbit polyclonal anti-βIII tubulin primary antibody (Catalogue # T2200) was sourced from Sigma-Aldrich, St. Louis, MO, USA, and secondary antibody (Alexa Fluor 568 goat anti-rabbit IgG) (Catalogue # A11011) was purchased from Thermo Fisher Scientific, Waltham, MA, USA. ProLong™ Gold Antifade Mountant with DAPI (Catalogue # P36935) was purchased from Thermo Fisher Scientific, Waltham, MA, USA. Citric acid monohydrate (Catalogue # LCS-CHEM-0068) was sourced from Sigma-Aldrich, supplied by Merck Life Science Pty Ltd., Bayswater, VIC, Australia, and trisodium citrate dihydrate (Catalogue # SL034-500G) was sourced from Chem-Supply Pty Ltd., Gillman, SA, Australia. A commercially available celery seed extract (High Strength Celery Seed, Nature’s Own, Brisbane, QLD, Australia) was purchased in capsule form.

### 2.5. Liquid Chromatography-Mass Spectrometry (LC–MS) Characterisation of the Celery Seed Extract

Commercially available Nature’s Own High Strength Celery Seed extract (4000 mg capsules) was used in this study. A qualitative LC–MS analysis was performed to provide chromatographic evidence supporting the presence of putative bioactive constituents within the extract. The contents of one capsule were dissolved in 10 mL methanol and filtered through a syringe filter. A 10 μL aliquot of the filtered extract was injected into an LC–MS system (Shimadzu LCMS-2020 single quadrupole mass spectrometer (Shimadzu Corporation, Kyoto, Japan)) for the qualitative analysis. Mass spectra were acquired over an *m/z* range of 100–1000, and major ions detected in the extract were compared with previously published mass spectral data for 3-n-butylphthalide (NBP) and related phthalides [35].

### 2.6. Induction of Diabetes

A combination of a HFD and a low-dose intraperitoneal (IP) STZ (30 mg/kg) injection was used to induce type 2 diabetes. Rats were fed the HFD for 8 weeks, followed by a single IP injection of STZ. On the day of STZ administration, rats were fasted for 4–6 h (7 a.m.–1 p.m.) prior to injection. This stepwise induction approach was adapted from previous research [20] for the development of type 2 diabetes. STZ (30 mg) was dissolved in 0.1 M sodium citrate buffer, and the pH was adjusted to 4.5 and utilised within 20 min of preparation [36]. A 1 mL aliquot of the STZ solution was administered to each animal using a 1 mL syringe and a 25 G needle. Four days post-STZ injection, the fasting blood glucose (FBG) was measured with an Accu-Check^®^ Performa II blood glucometer (Roche, Mannheim, Germany) to confirm the development of diabetes. Rats with blood glucose levels ≥250 mg/dL were considered diabetic and included in this study. Following diabetes induction, diabetic animals remained untreated for 4 weeks to allow for the development of diabetes-associated neuropathic changes prior to therapeutic intervention. The treatment began after 4 weeks of hyperglycaemia, and the rats were monitored over 4 weeks. The study duration, including the diabetes induction and subsequent treatment periods, was based on previous research demonstrating measurable neuropathic changes and therapeutic responses in rodent models of diabetic neuropathy [24,37,38].

### 2.7. Experimental Design

The experimental animals were randomly divided into 4 groups; the overall grouping of rats was as follows:

**Group A:** Healthy controls (*n* = 6): healthy rats were fed normal chow for the experimental duration.

**Group B:** Diabetic (*n* = 6): rats had diabetes induced using the HFD + STZ, after which they remained untreated throughout the experiment.

**Group C:** Diabetic + celery seed extract (*n* = 6): diabetic rats were treated with 100 mg/kg celery seed extract dissolved in a liquid paraffin (0.5 mL) every day for 4 weeks via IP injection [38].

**Group D:** Diabetic + Sitagliptin (*n* = 6): diabetic rats were administered sitagliptin 30 mg/kg, dissolved in distilled water, administered by oral gavage (0.5 mL) daily for 4 weeks [24].

All animals were kept on the HFD throughout the experiment, except for the healthy controls.

### 2.8. Fasting Blood Glucose Levels

The fasting blood glucose (FBG) levels for each rat were measured at baseline to confirm that all animals were at a similar blood glucose level, after diabetes induction, and at the end of the experiment. For the FBG levels, each rat was fasted for 5–6 h (morning fast 7 a.m.–1 p.m.). Blood glucose was measured using an Accu-Chek^®^ Performa II glucometer (Roche Diagnostics, Basel, Switzerland), with a measurable upper glucose limit of 33.3 mmol/L (600 mg/dL). Rats with blood glucose levels ≥250 mg/dL (13.9 mmol/L) were considered diabetic and included in this study.

### 2.9. Pain Perception Through Behavioural Tests

Pain perception was assessed simultaneously using the following behavioural tests on each testing day.

### 2.10. Tail Immersion Test

Thermal hyperalgesia was assessed using the tail immersion test, which measures thermal sensitivity to noxious stimuli. Thermal sensitivity was measured in all four groups at the end of the experiment. To perform this test, rats were restrained in a towel with their tails exposed and held for several min before the actual test to minimise stress during the procedure. The tail (1/3 portion) of each rat was immersed in cold (5–10 °C) or hot (52.5 ± 0.5 °C) water to measure the tail withdrawal response according to the protocol [39]. Tails were exposed to either temperature, and withdrawal latency (flicking response) or any other signs of struggle were observed; the cut-off time was set at 20–25 s to avoid injury.

Three consecutive tests were performed to determine the average latency value [40]. During the thermal sensitivity test, rats were closely monitored for any signs of discomfort, distress, or injury, including observing their behaviour, vocalisations, and physiological responses. A reduced tail-withdrawal latency was indicative of hyperalgesia [8].

### 2.11. Acetone Drop Test

Cold allodynia (as a measure of sensitivity to cool temperature) was determined from the hind paw using the acetone drop method. Briefly, a drop of acetone (a volatile liquid with a temperature of 15–20 °C) was sprayed onto the rat’s hind paws’ mid-plantar surface, and the paw withdrawal latencies were recorded. Each paw was tested once, with a 5-min interval between tests. Acetone was kept in small (25 mL) and easily handled containers.

To apply to the rat’s hind paw plantar surface, 25–30 µL of acetone was taken in a 1 mL syringe and carefully applied to the hind paw, and contact with other body parts was avoided. As soon as the acetone was applied, another researcher started the stopwatch to record the time of the first response (withdrawing/guarding, flicking, or licking) after application. To ensure the validity of the result, this test was performed on both hind paws with 5 × 5-min breaks between each. The mean of each test was calculated for every individual rat to quantify the results. A longer response duration (in s) was associated with allodynia [41].

### 2.12. Corneal Sensitivity

Corneal sensitivity was measured in unanesthetized rats using the Cochet–Bonnet filament Esthesiometer (Visionix, Pont-de-l’Arche, France). Testing began with the nylon filament fully extended to its maximum length (6 cm). The filament was gently touched to the cornea, and if the rat blinked (positive response), the filament length was recorded. If no blink occurred, the filament was shortened by 0.5 cm, and the test was repeated until a positive response was observed. To ensure accuracy, each eye was tested three times [42,43]. Due to the availability of animals that completed all endpoint assessments, post-treatment corneal sensitivity was analysed in a subset of animals, including the following group distribution (Diabetic *n* = 2, Diabetic + CSE *n* = 3, and Diabetic + sitagliptin *n* = 3).

### 2.13. Corneal Nerve Staining

At the conclusion of the experiment, the rats were euthanised, and their eyes were immediately enucleated and fixed in 2% paraformaldehyde for 1 h at room temperature. The corneas were carefully dissected and collected for whole-mount immunofluorescence staining. The excised corneas were rinsed in 0.1 M phosphate buffer (PB) prior to immunohistochemical processing.

The corneal tissue was immunostained using the protocol adopted from a previous report [44]. For staining, the corneas were processed free-floating in 48-well plates, with one cornea per well. The tissues were first rinsed in 0.1 M PB, pH 7.4 (2 × 5 min), then incubated in 1% sodium borohydride in 0.1 M PB for 30 min at room temperature to reduce autofluorescence. The corneas were then rinsed in 0.1 M PB (4 × 5 min) and then in 0.1 M Tris-buffered saline (TS), pH 7.6 (2 × 5 min). The corneas were incubated in 0.5% bovine serum albumin (BSA) in 0.1 M TS for 30 min at room temperature to block. The corneas were incubated with the rabbit anti-βIII-tubulin primary antibody (Sigma-Aldrich, Australia, Cat. No. T2200-200UL, stock concentration 0.4–1.0 mg/mL) prepared in 0.1% BSA and 0.25% Triton X-100 in 0.1 M TS, in a 1:500 dilution. Primary antibody incubation was carried out for 72 h at 4 °C on a shaker, with the epithelial side facing upward. After primary antibody incubation, the corneas were rinsed in 0.1 M TS (3 × 5 min) and then incubated for 2 h at room temperature with goat anti-rabbit IgG (H + L), cross-adsorbed, Alexa Fluor 568 secondary antibody (Thermo Fisher Scientific; Cat. No. A11011) diluted 1:500 in 0.1% BSA in 0.1 M TS. From this step onward, the tissues were protected from light. The corneas were then rinsed in 0.1 M TS (3 × 5 min) and finally in 0.1 M PB (3 × 5 min).

For mounting, the corneas were transferred to 0.05 M PB, and four radial incisions were made to assist in flattening the tissue into a cloverleaf configuration. Each cornea was mounted epithelial side up on a glass slide and carefully flattened. The slides were coverslipped with ProLong™ Gold Antifade mounting medium (Thermo Fisher Scientific) and allowed to cure for 24–48 h in the dark. Mounted slides were stored in light-protected containers at −20 °C until imaging.

### 2.14. Confocal Image Acquisition

One eye from each animal was imaged using a scanning confocal microscope (LSM 980, Olympus, Tokyo, Japan) with a 20x air objective lens. Positive fluorescence signals were captured using a 561 nm laser. Z-stacks were collected from each quadrant of βIII-tubulin immunolabeled corneas. Four images per cornea were acquired to ensure the same area was measured for corneal nerve parameters. Images were taken from the peripheral region of the cornea, defined as areas adjacent to the limbus and outside the central zone, based on a visual assessment during image acquisition. Maximum-intensity projections were generated from these Z-stacks using Zen Black 3.12 software. After conversion to 8-bit grayscale, images were quantified for nerve fibre parameters using NoBS and the skeleton analyser, a plugin in the ImageJ software (version ImageJ 1.54f) [45]. These parameters include the total length of nerve fibres, which comprises the sum of all fibres present in the image (mm/mm^2^). Density measures the nerve occupancy and is expressed as a percentage of pixels in each image occupied by the intra-epithelial cornea basal nerves (ICBNs), and nerve tortuosity [46].

### 2.15. Statistical Analysis

Statistical analyses were conducted using GraphPad Prism version 9.5.1. All outcomes were presented as mean ± SEM, and the data were subjected to a t-test and one-/two-way analysis of variance (ANOVA) where appropriate, followed by Tukey post hoc comparisons. Statistical analyses were performed using individual rats as the experimental unit. The sample size was calculated based on the previous literature [20] using a similar diabetic model and rat strain. G*Power software, version 3.1.9.4, was used to estimate the required sample size, resulting in 6 animals per group for the analysis. All the analysis and outcome assessments were performed in a blinded manner where possible.

## 3. Results

### 3.1. LC–MS Characterisation of Celery Seed Extract

**Figure 1:**
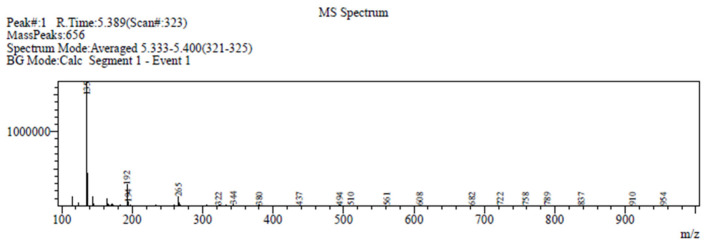
Representative mass spectrum of Peak #1 obtained from a qualitative LC–MS analysis of the celery seed extract. The spectrum was acquired at RT 5.389 min (average scan range 5.333–5.400 min. Prominent ions were observed at *m/z* 135 and *m*/*z* 192. Comparison with previously published mass spectral data suggested the tentative presence of NBP-related phthalides in the extract.

NBP has a molecular mass of 190.10 Da and can undergo characteristic fragmentation during electrospray ionisation. Previous studies have reported that cleavage of the n-butyl side chain generates a fragment ion at *m*/*z* 135 [35]. Consistent with this report, our LC–MS analysis detected a prominent ion at a retention time of approximately 6.0 min with a corresponding fragment at *m*/*z* 135, representing approximately 50% relative abundance (Figure 1). This fragmentation pattern is consistent with the tentative presence of NBP in the celery seed extract. To further support the qualitative LC–MS characterisation of the celery seed extract, the corresponding PDA and LC–MS chromatograms are provided in Supplementary Figure S1.

### 3.2. Blood Glucose

**Figure 2:**
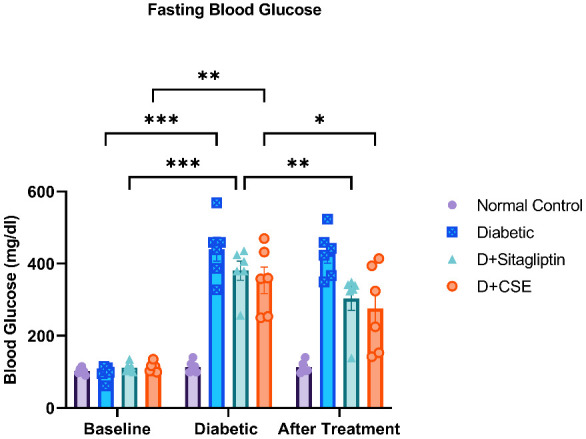
Fasting blood glucose in normal, diabetic, diabetic + sitagliptin (D + sitagliptin), and diabetic + celery seed extract (D + CSE) rats was measured at baseline, following diabetes induction, and after treatment. Data are presented as mean ± SEM. A statistical analysis was performed using repeated measures two-way ANOVA followed by Tukey’s multiple comparisons test. *n* = 6 animals per group. * *p* < 0.05, ** *p* < 0.002, *** *p* < 0.001.

The FBG levels were measured in all 24 rats at baseline and were similar across all animals. After diabetes induction (*n* = 18), the blood glucose levels were significantly higher in diabetic rats (337.3 ± 25.8 mg/dL) than in their respective baseline values (107.1 ± 3.8 mg/dL). Diabetic rats were subsequently divided into two treatment groups (*n* = 6/group). Following treatment, the blood glucose levels remained higher in untreated diabetic rats (427.5 ± 1.4 mg/dL), whereas both sitagliptin (282.6 ± 2.4 mg/dL) and celery seed extract (301.8 ± 2.3 mg/dL) significantly reduced the FBG compared with those for untreated diabetic rats (Figure 2).

### 3.3. Tail Immersion Test

**Figure 3:**
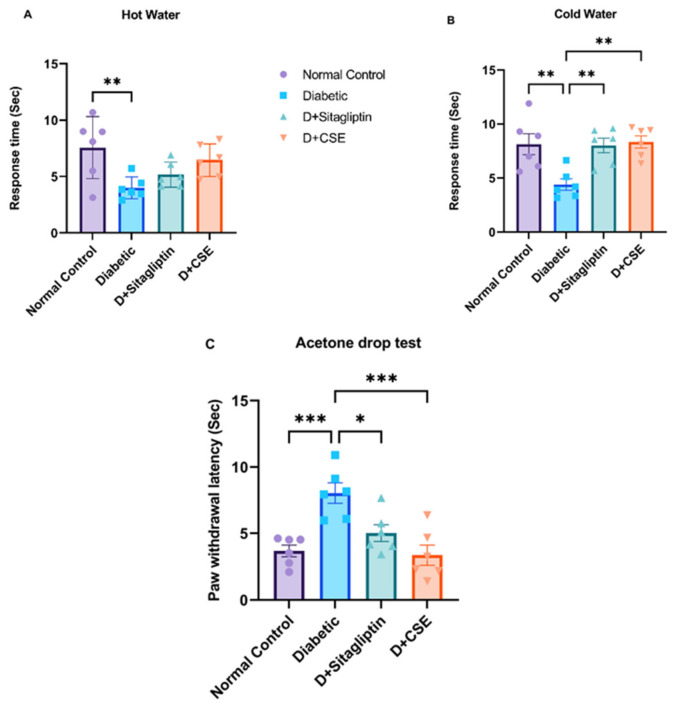
Response latency in the hot-water (**A**), cold-water (**B**), and acetone drop tests (**C**) in normal, diabetic, diabetic + sitagliptin (D + sitagliptin), and diabetic + celery seed extract (D + CSE) rats. Data are presented as mean ± SEM. A statistical analysis was performed using one-way ANOVA followed by Tukey’s multiple comparisons test. *n* = 6 animals per group. * *p* < 0.05, ** *p* < 0.002, *** *p* < 0.001.

Diabetic rats showed a significant reduction in tail-withdrawal latency in both the hot- and cold-water tail-immersion tests (Figure 3A,B) compared with normal controls. In the hot-water immersion test, withdrawal latency decreased from 7.57 ± 1.12 s for normal rats to 3.99 ± 0.39 s for diabetic rats (*p* < 0.05). Similarly, in the cold-water immersion test, diabetic rats showed a significant reduction in latency (normal: 8.13 ± 0.96 s; diabetic: 4.39 ± 0.52 s; *p* < 0.05), indicating the development of thermal hyperalgesia. Following treatment, both sitagliptin and celery seed extract significantly increased withdrawal latency for the cold immersion test (diabetic + sitagliptin: 8.3 ± 0.6 s; diabetic + celery seed extract: 8.02 ± 0.6 s; *p* < 0.05 vs. diabetic). However, no significant improvement was observed for the hot-water immersion test, although a trend toward increased withdrawal latency with celery seed extract was noted (*p* = 0.09).

### 3.4. Acetone Drop Test

The response latency in the acetone drop test was significantly longer for diabetic rats than for normal rats, indicating decreased cold sensitivity, consistent with diabetic neuropathy (Figure 3C). The mean paw withdrawal latency of diabetic rats was 8.0 ± 0.7 s, while that of normal rats was 3.6 ± 0.4 s (*p* < 0.05). Treatment with sitagliptin (5.0 ± 0.6 s) and celery seed extract (3.4 ± 0.7 s, *p* < 0.05 for both), significantly reduced the response latency compared with untreated diabetic rats.

### 3.5. Corneal Sensitivity

**Figure 4:**
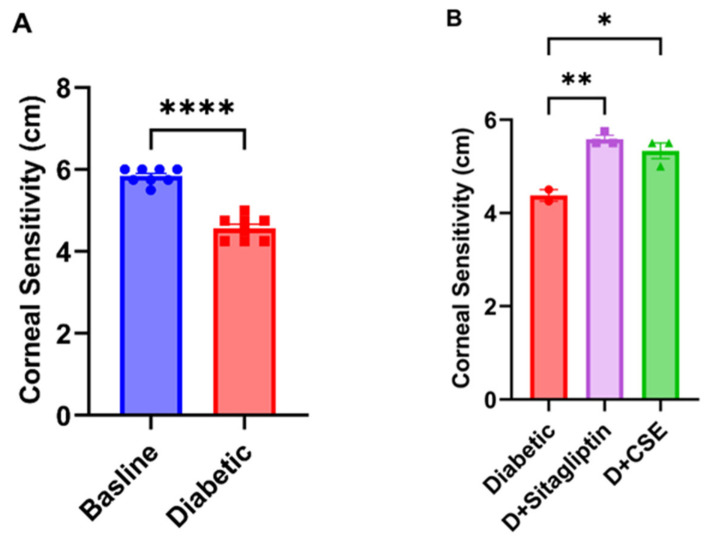
Corneal sensitivity threshold at baseline and remeasured in the same animals after diabetes induction in diabetic + sitagliptin (D + sitagliptin), and diabetic + celery seed extract (D + CSE) rats. Corneal sensitivity was measured in diabetic rats (*n* = 8) compared with baseline (*n* = 8) (paired t-test (**A**), and after treatment, the groups’ sensitivity (D + sitagliptin; *n* = 3; D + CSE; *n* = 3) was compared with that of untreated diabetic rats (*n* = 2) (one-way ANOVA with Tukey’s post hoc test (**B**). Data are presented as mean ± SEM. * *p* < 0.05, ** *p* < 0.01, **** *p* < 0.0001.

Corneal sensitivity was measured using a Cochet–Bonnet esthesiometer at baseline in eight rats before dietary intervention or diabetes induction and after diabetes development. Diabetes significantly decreased corneal sensitivity compared to the baseline. The mean corneal sensitivity values at baseline were 5.8 ± 0.1 cm vs. 4.5 ± 0.1 cm for diabetic rats; *p* < 0.05 (Figure 4A). Following diabetes induction, rats were allocated into three groups: untreated diabetic rats (*n* = 2), sitagliptin-treated diabetic rats (diabetic + sitagliptin, *n* = 3), and celery seed-treated diabetic rats (diabetic + celery seed extract, *n* = 3). Corneal sensitivity was measured again at the end of the experiment. One-way ANOVA among the groups demonstrated a significant effect for both treatments. In the post hoc analysis, both celery seed extract and sitagliptin showed the potential to improve corneal sensitivity compared to untreated diabetic rats (diabetic + sitagliptin: 5.5 ± 0.1; diabetic + celery seed extract: 5.3 ± 0.1; untreated diabetic: 4.3 ± 0.1; *p* < 0.05) (Figure 4B). Although the observed effects were statistically significant, the small sample size limits the robustness of these findings, and they should be considered as preliminary.

### 3.6. Corneal Nerve Analyses

**Figure 5:**
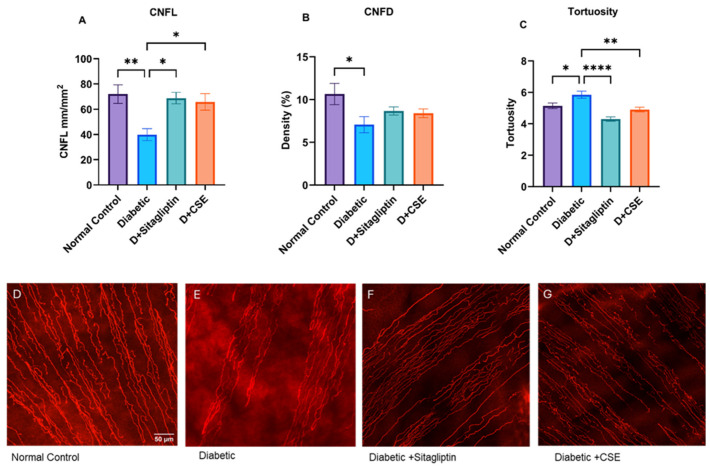
Analysis of the peripheral corneal nerves in normal, diabetic (D), diabetic + sitagliptin (D + sitagliptin), and diabetic + celery seed extract (D + CSE) groups. Panels (**A**–**C**) show a quantitative analysis of CNFL (**A**), CNFD (**B**), and nerve tortuosity (**C**), respectively. Panels (**D**–**G**) show representative βIII-tubulin–positive peripheral corneal nerve images from (**D**) normal, (**E**) diabetic, (**F**) D + sitagliptin, and (**G**) D + CSE groups. Red fluorescence represents βIII-tubulin immunolabelling of the corneal nerve fibres. Scale bar = 50 µm. Data are presented as mean ± SEM. A statistical analysis was performed using one-way ANOVA followed by Tukey’s multiple comparisons test. *n* = 6 animals per group. Statistical significance was considered at *p* < 0.05. Asterisks denote significant group differences * *p* < 0.05, ** *p* < 0.01, **** *p* < 0.0001.

Measurements of the corneal nerve fibre parameters are shown in Figure 5. The CNFL differed significantly among the experimental groups. Diabetic rats showed a significant reduction in the CNFL compared with normal rats (mean ± SEM: diabetic: 39.8 ± 4.7 mm/mm^2^; normal: 71.7 ± 7.3 mm/mm^2^, *p* < 0.05). Treatment with sitagliptin and celery seed extract significantly increased the CNFL compared with untreated diabetic rats (diabetic: 39.8 ± 4.7 mm/mm^2^; diabetic + sitagliptin: 68.8 ± 4.5; diabetic + celery seed extract: 65.8 ± 6.5 mm/mm^2^, *p* < 0.05; Figure 5A). Similarly, the corneal nerve fibre density (CNFD) was significantly reduced in diabetic rats compared with normal rats (mean ± SEM: diabetic: 7.1 ± 0.9%; normal: 10.6 ± 1.2%, *p* < 0.05). Neither sitagliptin nor celery seed extract significantly improved the CNFD compared with diabetic rats (diabetic + sitagliptin: 8.6 ± 0.4%; diabetic + celery seed extract: 8.4 ± 0.5%; diabetic: 7.1 ± 0.9%; Figure 5B). Corneal nerve tortuosity was significantly increased in diabetic rats compared with normal controls (mean ± SEM: diabetic: 5.8 ± 0.2 vs. normal: 5.1 ± 0.1; *p* < 0.05). Both sitagliptin and celery seed extract significantly reduced tortuosity relative to diabetic groups (diabetic + sitagliptin: 4.3 ± 0.1%; diabetic + celery seed extract: 4.9 ± 0.1%; diabetic: 5.8 ± 0.2%, *p* < 0.05; Figure 5C). Representative corneal confocal images from normal, untreated diabetic, and treated groups are shown in Figure 5D–G.

## 4. Discussion

This study investigated the effects of celery seed extract and sitagliptin on diabetes-induced peripheral neuropathy and corneal nerve alterations in an HFD + STZ-induced type 2 diabetic rat model. The HFD + STZ-induced diabetic rats exhibited marked hyperglycaemia, together with structural and functional features consistent with peripheral neuropathy, including significant reductions in the CNFL and CNFD, and increased nerve tortuosity. Similarly, altered thermal responses in the hot- and cold-water tests, and a delayed response in the acetone drop test were observed, implicating sensory dysfunction. Both treatments significantly lowered the blood glucose levels, increased the CNFL, reduced corneal nerve tortuosity, and partially improved behavioural sensory measures. Significantly higher blood glucose levels in diabetic rats compared to their baseline measurement confirmed the successful induction of diabetes in the HFD + STZ group, consistent with previous studies [47]. Hyperglycaemia was significantly reduced in rats treated with both sitagliptin and celery seed extract; however, the glucose levels in treated groups remained higher than those of normal rats. These findings suggest that both treatments partially restored blood glucose levels to normal but did not completely normalise the hyperglycaemic state.

The reduction in the CNFL and CNFD observed in diabetic rats is consistent with both clinical and experimental studies showing that diabetes is associated with early small nerve fibre damage, which occurs during DPN [48,49,50]. Corneal nerves are among the earliest small fibres affected in diabetes and are highly sensitive to metabolic dysfunction, making them indicators of peripheral neuropathy [51,52]. Hyperglycaemia-induced oxidative stress, mitochondrial dysfunction, chronic inflammation, and impaired neurotrophic support are recognised contributors to diabetic neuropathy and may contribute to the corneal nerve degeneration observed in the present study [53,54]. These pathological processes can impair axonal transport, disrupt neuronal energy metabolism, and promote progressive nerve fibre loss, ultimately leading to reductions in corneal nerve morphology [55]. The findings of the present study suggest that corneal nerve alterations indicate the development of diabetic neuropathy in the HFD + STZ model [56]. The reduction in the CNFL appeared more pronounced and more responsive to treatment than that in the CNFD, suggesting that nerve fibre length may be a more sensitive marker of early degradation and regeneration than nerve density. This is consistent with previous studies showing that the CNFL is a sensitive CCM-derived marker for detecting small fibre damage, monitoring diabetic neuropathy progression, and identifying nerve repair or regeneration [57]. In the present study, the significant improvement in the CNFL, despite no significant change in the CNFD, may indicate partial structural repair rather than complete restoration of the corneal nerve.

Treatment with both sitagliptin and celery seed extract significantly increased the CNFL compared with untreated diabetic rats, whereas the CNFD did not significantly improve. Similar findings have been reported in clinical studies of DPN using corneal confocal microscopy, in which therapeutic intervention resulted in a significant improvement in the CNFL, while the CNFD showed a non-significant recovery [58]. Clinical studies have shown that corneal nerve morphology can improve with improved glycaemic control [51], and animal studies have also suggested that corneal nerve loss can be reversible under favourable metabolic conditions [59]. In the present study, although the blood glucose levels remained higher than in the normal control rats, both sitagliptin and celery seed extract significantly improved the CNFL following treatment. These findings are consistent with previous studies, which have suggested that complete normalisation of glycaemia may not be necessary to initiate structural nerve repair, and that partial improvement in the metabolic environment may be sufficient to promote early corneal nerve regeneration [60].

Corneal nerve tortuosity was increased in diabetic rats and reduced after treatment with sitagliptin or celery seed extract. Increased tortuosity has been reported in diabetic patients with neuropathy, where it has been identified as a marker of nerve degeneration and regeneration, or of altered nerve fibre function [61,62]. Experimental studies have suggested that chronic hyperglycaemia promotes oxidative stress, mitochondrial dysfunction, inflammatory signalling, and impaired axonal transport, all of which disrupt normal axonal maintenance and may contribute to abnormal nerve fibre architecture and increased tortuosity [63] In DPN, metabolic disturbances, oxidative stress, and impaired axonal transport may lead to abnormal nerve morphology and increased curvature of nerve fibres. Therefore, the reduction in tortuosity observed after treatment with sitagliptin and celery seed extract may indicate improved corneal nerve integrity and a shift towards regeneration, which is necessary to restore corneal health.

Corneal sensitivity was also significantly reduced in diabetic rats, indicating dysfunction of the small sensory fibres innervating the cornea. Reduced corneal sensitivity is a recognised feature of diabetic neuropathy and has been reported in both patients and diabetic animal models [56], together with corneal nerve loss. In the present study, reduced corneal sensitivity paralleled reductions in the CNFL and CNFD, supporting a close relationship between corneal nerve structure and function. Previous studies have demonstrated that corneal nerve morphology and corneal sensitivity thresholds are potential surrogate markers for assessing diabetic neuropathy [64]. Both sitagliptin and celery seed extract showed the potential to improve corneal sensitivity compared with untreated diabetic rats, indicating partial restoration of corneal sensory function. However, the sensitivity threshold did not return to the same level as that of the normal control group. This recovery in corneal sensitivity is likely related to the increased CNFL and restoration of corneal nerve function. Similar findings have been reported in previous animal studies, in which interventions that improved corneal nerve morphology also improved corneal sensitivity [65]. The differential response of corneal nerve parameters is consistent with a previous report [66] in which corneal nerve regeneration was not uniform across all parameters, with relatively greater changes in the CNFL than in the CNFD. The increase in the CNFL reflects early nerve regeneration, while the lack of improvement in the CNFD suggests that nerve regeneration is not complete. Although both treatments promote early nerve regeneration, full structural restoration has not yet occurred. This incomplete recovery may contribute to partial improvement in corneal sensory function.

Sensory abnormalities were also observed in diabetic rats, including thermal hyperalgesia, as evidenced by increased pain sensitivity. These findings support the presence of diabetic neuropathy in this model and are consistent with previous studies using the HFD + STZ model, which demonstrated thermal hyperalgesia in the early phase of diabetic neuropathy [67]. In the present study, the diabetic group showed a significant reduction in tail withdrawal latency to noxious hot or cold stimuli compared to the normal control group, indicating increased sensitivity and the development of thermal hyperalgesia. Treatment with sitagliptin and celery seed extract significantly increased withdrawal latency in the cold-water test. However, neither treatment significantly increased it in the hot-water test. In contrast to the hot- and cold-water tests, the acetone drop test showed a significantly delayed response latency, indicating cold allodynia consistent with previous studies [68]. Both sitagliptin and celery seed extract treatment reduced the response time in the acetone drop test compared with untreated diabetic rats, suggesting a restoration of small fibre function.

The beneficial effects of sitagliptin are not only attributed to its glucose-lowering capacity but to its anti-inflammatory and neuroprotective properties. Previous studies have reported its anti-inflammatory and neuroprotective effects in diabetic neuropathy models [18,69]. Sitagliptin, an approved drug for the treatment of type 2 diabetes, inhibits dipeptidyl peptidase-4. It increases glucagon-like peptide-1 (GLP-1) signalling and reduces oxidative stress, inflammatory cytokine production, and neuronal apoptosis. These actions could help to preserve small sensory fibres and to reduce neuropathic pain [70]. Sitagliptin is well-tolerated and has been used clinically, although some adverse effects, including headaches, gastrointestinal symptoms, and rare cases of pancreatitis, have been reported in previous studies [71].

Improved thermal sensitivity, including hyperalgesia and cold allodynia, was observed in diabetic rats following treatment with celery seed extract. Additionally, corneal sensitivity and other corneal nerve fibre parameters were ameliorated in celery seed extract-treated diabetic rats. Celery seed extract contains a high concentration of bioactive phytochemicals and has been reported to have antioxidant, anti-inflammatory, and neuroprotective properties [31]. Among these constituents, *N*-butylphthalide (NBP) has been extensively studied in experimental models of neurological disease, where it has been shown to improve mitochondrial function, to reduce oxidative stress, to suppress neuroinflammatory signalling, and to preserve neuronal survival and synaptic function [72,73]. Celery seed extract has been reported to be well tolerated in a human study, with no significant adverse effects observed during the supplementation period [74], supporting its potential safety and translational relevance as a therapeutic intervention. In the present study, NBP was identified using liquid chromatography–mass spectrometry (LC–MS), providing evidence of the tentative presence of a neuroprotective compound (NBP) in the administered extract. These improvements in sensory responses observed after celery seed extract treatment could therefore likely be due to a combination of improved glycaemic control and direct neuroprotective effects. Celery seed extract has generally been reported to have a favourable safety profile in both experimental and clinical studies, with only mild adverse effects, such as gastrointestinal discomfort or abdominal distension, reported in some cases [75,76]. No obvious adverse effects related to celery seed extract treatment were observed during the present study; however, dedicated toxicity and long-term safety assessments were not performed.

In the present study, corneal nerve analysis was performed on the peripheral regions of ICBNs, whereas the central cornea and inferior whorl were not specifically evaluated, reflecting a limitation of this study. Previous studies have demonstrated that regional differences in corneal nerves may occur in diabetes, particularly in the inferior whorl, which is a specialised region of ICBNs and a sensitive marker of small nerve fibre damage in DPN progression. [77]. Studies have also reported loss of corneal nerves in both the central cornea and the inferior whorls in DPN, suggesting that diabetes affects multiple regions of ICBNs [78]. Therefore, future studies incorporating standardised regional mapping of central, peripheral, and inferior whorl nerve fibres may provide a more comprehensive understanding of diabetes-induced corneal nerve damage and of regeneration after treatment. 

Both sitagliptin and celery seed extract improved several neuropathy-related parameters in the present study. Sitagliptin may have greater translational potential because of its established clinical use and well-characterised pharmacological profile in type 2 diabetes [70]. Celery seed extract also demonstrated comparable beneficial effects, being a natural phototherapeutic compound, which may be attributed to its antioxidant and anti-inflammatory constituents, such as NBP [79], suggesting its therapeutic potential. Although both treatments have demonstrated beneficial effects on corneal nerve architecture and DPN-related functional outcomes, sitagliptin may have greater translational potential for clinical application due to its established pharmacokinetic profile and existing use in type 2 diabetes.

### Limitations

An important limitation of this study is its small sample size for the post-treatment assessment of corneal sensitivity. Given the small sample size available for post-treatment corneal sensitivity measurements, these findings should be considered preliminary and require confirmation in larger cohorts. Additionally, the vehicle control group receiving liquid paraffin (solvent for celery seed extract) was not included in this study. Therefore, the observed effects should be considered preliminary and require further confirmation with a larger sample size and the incorporation of a vehicle control group.

## 5. Conclusions

The present study demonstrated that experimental type 2 diabetes is associated with corneal nerve loss and sensory dysfunction, as evidenced by reductions in the CNFL and CNFD and alterations in corneal sensitivity. Impaired behavioural responses were also observed in the type 2 diabetic rat model. Treatment with sitagliptin and celery seed extract improved several structural and functional measures of neuropathy, suggesting their potential neuroprotective effect. The role of sitagliptin and celery seed extract in corneal nerve parameters and corneal sensory function has not been sufficiently studied to date. The results of the current study suggest that both sitagliptin and celery seed extract have potential as therapeutic agents for diabetic neuropathy and corneal nerve damage, and that corneal sensitivity and corneal nerve fibres may be useful for monitoring therapeutic response in future studies. Future studies with larger sample sizes should incorporate detailed physicochemical profiling of celery seed extract, evaluate dose-response relationships, and investigate the molecular mechanisms underlying both interventions and their neuroprotective effects, including pathways related to oxidative stress, inflammation, mitochondrial dysfunction, and neurotrophic signalling.

## Data Availability

Data are available from the corresponding author upon reasonable request.

## Supplementary Materials

The following supporting information can be downloaded at: https://www.mdpi.com/article/10.3390/nu18142243/s1, Figure S1: LC–MS Characterisation of Celery Seed Extract.

## Abbreviations

The following abbreviations are used in this manuscript:

DPN	Diabetic peripheral neuropathy
CNFL	Corneal nerve fibre length
CNFD	Corneal nerve fibre density
CSE	Celery seed extract
SD	Sprague Dawley
HFD	High-fat diet
STZ	Streptozotocin

## References

[1] Genitsaridi I., Salpea P., Salim A., Sajjadi S.F., Tomic D., James S., Thirunavukkarasu S., Issaka A., Chen L., Basit A. (2026). 11th edition of the IDF Diabetes Atlas: Global, regional, and national diabetes prevalence estimates for 2024 and projections for 2050. Lancet Diabetes Endocrinol..

[2] Saeedi P., Petersohn I., Salpea P., Malanda B., Karuranga S., Unwin N., Colagiuri S., Guariguata L., Motala A.A., Ogurtsova K. (2019). Global and regional diabetes prevalence estimates for 2019 and projections for 2030 and 2045: Results from the International Diabetes Federation Diabetes Atlas. Diabetes Res. Clin. Pract..

[3] Carmichael J., Fadavi H., Ishibashi F., Shore A.C., Tavakoli M. (2021). Advances in Screening, Early Diagnosis and Accurate Staging of Diabetic Neuropathy. Front. Endocrinol..

[4] Dhage S., Ferdousi M., Adam S., Ho J.H., Kalteniece A., Azmi S., Alam U., Ponirakis G., Petropoulos I., Atkinson A.J. (2021). Corneal confocal microscopy identifies small fibre damage and progression of diabetic neuropathy. Sci. Rep..

[5] Pang L., Lian X., Liu H., Zhang Y., Li Q., Cai Y., Ma H., Yu X. (2020). Understanding Diabetic Neuropathy: Focus on Oxidative Stress. Oxid. Med. Cell. Longev..

[6] Yagihashi S., Mizukami H., Sugimoto K. (2011). Mechanism of diabetic neuropathy: Where are we now and where to go?. J. Diabetes Investig..

[7] Kaeidi A., Esmaeili-Mahani S., Sheibani V., Abbasnejad M., Rasoulian B., Hajializadeh Z., Afrazi S. (2011). Olive (*Olea europaea* L.) leaf extract attenuates early diabetic neuropathic pain through prevention of high glucose-induced apoptosis: In vitro and in vivo studies. J. Ethnopharmacol..

[8] Yadav S.K., Nagori B.P., Desai P.K. (2014). Pharmacological characterization of different fractions of Calotropis procera (Asclepiadaceae) in streptozotocin induced experimental model of diabetic neuropathy. J. Ethnopharmacol..

[9] Tesfaye S., Selvarajah D. (2009). The Eurodiab study: What has this taught us about diabetic peripheral neuropathy?. Curr. Diabetes Rep..

[10] Jang H.N., Oh T.J. (2023). Pharmacological and Nonpharmacological Treatments for Painful Diabetic Peripheral Neuropathy. Diabetes Metab. J..

[11] Khdour M.R. (2020). Treatment of diabetic peripheral neuropathy: A review. J. Pharm. Pharmacol..

[12] Petropoulos I.N., Ponirakis G., Khan A., Almuhannadi H., Gad H., Malik R.A. (2018). Diagnosing Diabetic Neuropathy: Something Old, Something New. Diabetes Metab. J..

[13] Chen X., Graham J., Dabbah M.A., Petropoulos I.N., Ponirakis G., Asghar O., Alam U., Marshall A., Fadavi H., Ferdousi M. (2015). Small nerve fiber quantification in the diagnosis of diabetic sensorimotor polyneuropathy: Comparing corneal confocal microscopy with intraepidermal nerve fiber density. Diabetes Care.

[14] Guerrero-Moreno A., Baudouin C., Melik Parsadaniantz S., Réaux-Le Goazigo A. (2020). Morphological and Functional Changes of Corneal Nerves and Their Contribution to Peripheral and Central Sensory Abnormalities. Front. Cell. Neurosci..

[15] Mansoor H., Tan H.C., Lin M.T., Mehta J.S., Liu Y.C. (2020). Diabetic Corneal Neuropathy. J. Clin. Med..

[16] Reed M.J., Meszaros K., Entes L.J., Claypool M.D., Pinkett J.G., Gadbois T.M., Reaven G.M. (2000). A new rat model of type 2 diabetes: The fat-fed, streptozotocin-treated rat. Metabolism.

[17] Barrière D.A., Noll C., Roussy G., Lizotte F., Kessai A., Kirby K., Belleville K., Beaudet N., Longpré J.M., Carpentier A.C. (2018). Combination of high-fat/high-fructose diet and low-dose streptozotocin to model long-term type-2 diabetes complications. Sci. Rep..

[18] Yin R., Xu Y., Wang X., Yang L., Zhao D. (2022). Role of Dipeptidyl Peptidase 4 Inhibitors in Antidiabetic Treatment. Molecules.

[19] Zhou T., Lee A., Lo A.C.Y., Kwok J. (2022). Diabetic Corneal Neuropathy: Pathogenic Mechanisms and Therapeutic Strategies. Front. Pharmacol..

[20] Coppey L., Davidson E., Shevalye H., Obrosov A., Torres M., Yorek M.A. (2020). Progressive loss of corneal nerve fibers and sensitivity in rats modeling obesity and type 2 diabetes is reversible with omega-3 fatty acid intervention: Supporting cornea analyses as a marker for peripheral neuropathy and treatment. Diabetes Metab. Syndr. Obes..

[21] Shawky L.M., Morsi A.A., El Bana E., Hanafy S.M. (2019). The Biological Impacts of Sitagliptin on the Pancreas of a Rat Model of Type 2 Diabetes Mellitus: Drug Interactions with Metformin. Biology.

[22] Mani V., Arfeen M. (2024). In Vivo and Computational Studies on Sitagliptin’s Neuroprotective Role in Type 2 Diabetes Mellitus: Implications for Alzheimer’s Disease. Brain Sci..

[23] Sharma A.K., Sharma A., Kumari R., Kishore K., Sharma D., Srinivasan B.P., Sharma A., Singh S.K., Gaur S., Jatav V.S. (2012). Sitagliptin, sitagliptin and metformin, or sitagliptin and amitriptyline attenuate streptozotocin-nicotinamide induced diabetic neuropathy in rats. J. Biomed. Res..

[24] Kelany M.E., Hakami T.M., Omar A.H., Abdallah M.A. (2016). Combination of Sitagliptin and Insulin against Type 2 Diabetes Mellitus with Neuropathy in Rats: Neuroprotection and Role of Oxidative and Inflammation Stress. Pharmacology.

[25] Tiwari R., Siddiqui M.H., Mahmood T., Bagga P., Ahsan F., Shamim A. (2019). Herbal Remedies: A Boon for Diabetic Neuropathy. J. Diet. Suppl..

[26] Zhang Y., Yao L., Lyu Y., Tang Z., Liu X., Duan X. (2025). From bench to bedside: Therapeutic potential of natural antioxidants in diabetic neuropathy. Front. Cell Dev. Biol..

[27] Shayani Rad M., Moohebati M., Mohajeri S.A. (2023). Beneficial effects of celery seed extract (*Apium graveolens*), as a supplement, on anxiety and depression in hypertensive patients: A randomized clinical trial. Inflammopharmacology.

[28] Cho B.O., Che D.N., Shin J.Y., Kang H.J., Kim J.H., Jang S.I. (2020). Anti-obesity effects of enzyme-treated celery extract in mice fed with high-fat diet. J. Food Biochem..

[29] Kooti W., Daraei N. (2017). A Review of the Antioxidant Activity of Celery (*Apium graveolens* L.). J. Evid. Based Complement. Altern. Med..

[30] Mans K., Aburjai T. (2019). Accessing the hypoglycemic effects of seed extract from celery (*Apium graveolens*) in alloxan-induced diabetic rats. J. Pharm. Res. Int..

[31] Hedayati N., Bemani Naeini M., Mohammadinejad A., Mohajeri S.A. (2019). Beneficial effects of celery (*Apium graveolens*) on metabolic syndrome: A review of the existing evidences. Phytother. Res..

[32] Tan T.Y.C., Lim X.Y., Norahmad N.A., Chanthira Kumar H., Teh B.P., Lai N.M., Syed Mohamed A.F. (2023). Neurological Applications of Celery (*Apium graveolens*): A Scoping Review. Molecules.

[33] Luo R., Wangqin R., Zhu L., Bi W. (2019). Neuroprotective mechanisms of 3-n-butylphthalide in neurodegenerative diseases. Biomed. Rep..

[34] Kaur M., Misra S., Swarnkar P., Patel P., Das Kurmi B., Das Gupta G., Singh A. (2023). Understanding the role of hyperglycemia and the molecular mechanism associated with diabetic neuropathy and possible therapeutic strategies. Biochem. Pharmacol..

[35] Diao X., Deng P., Xie C., Li X., Zhong D., Zhang Y., Chen X. (2013). Metabolism and pharmacokinetics of 3-n-butylphthalide (NBP) in humans: The role of cytochrome P450s and alcohol dehydrogenase in biotransformation. Drug Metab. Dispos..

[36] Goyal S.N., Reddy N.M., Patil K.R., Nakhate K.T., Ojha S., Patil C.R., Agrawal Y.O. (2016). Challenges and issues with streptozotocin-induced diabetes—A clinically relevant animal model to understand the diabetes pathogenesis and evaluate therapeutics. Chem. Biol. Interact..

[37] Hossain M.J., Kendig M.D., Letton M.E., Morris M.J., Arnold R. (2022). Peripheral Neuropathy Phenotyping in Rat Models of Type 2 Diabetes Mellitus: Evaluating Uptake of the Neurodiab Guidelines and Identifying Future Directions. Diabetes Metab. J..

[38] Tashakori-Sabzevar F., Ramezani M., Hosseinzadeh H., Parizadeh S.M.R., Movassaghi A.R., Ghorbani A., Mohajeri S.A. (2016). Protective and hypoglycemic effects of celery seed on streptozotocin-induced diabetic rats: Experimental and histopathological evaluation. Acta Diabetol..

[39] Saini A.K., Kumar H.S.A., Sharma S.S. (2007). Preventive and curative effect of edaravone on nerve functions and oxidative stress in experimental diabetic neuropathy. Eur. J. Pharmacol..

[40] Zhou Q., Bao Y., Zhang X., Zeng L., Wang L., Wang J., Jiang W. (2014). Optimal interval for hot water immersion tail-flick test in rats. Acta Neuropsychiatr..

[41] Khan A., Shal B., Khan A.U., Baig M.W., Haq I.U., Khan S. (2023). Withametelin, a steroidal lactone, isolated from datura innoxa attenuates STZ-induced diabetic neuropathic pain in rats through inhibition of NF-kB/MAPK signaling. Food Chem. Toxicol..

[42] Davidson E.P., Coppey L.J., Holmes A., Yorek M.A. (2012). Changes in corneal innervation and sensitivity and acetylcholine-mediated vascular relaxation of the posterior ciliary artery in a type 2 diabetic rat. Investig. Ophthalmol. Vis. Sci..

[43] Akowuah P.K., Hargrave A., Rumbaut R.E., Burns A.R. (2021). Dissociation between Corneal and Cardiometabolic Changes in Response to a Time-Restricted Feeding of a High Fat Diet. Nutrients.

[44] Hegarty D.M., Hermes S.M., Morgan M.M., Aicher S.A. (2018). Acute hyperalgesia and delayed dry eye after corneal abrasion injury. Pain Rep..

[45] Tuck H., Park M., Carnell M., Machet J., Richardson A., Jukic M., Di Girolamo N. (2021). Neuronal-epithelial cell alignment: A determinant of health and disease status of the cornea. Ocul. Surf..

[46] Machet J., Park M., Richardson A., Carnell M., Mouat M.A., Smith N.J., Turner N., Cochran B.J., Rye K.A., Di Girolamo N. (2023). Type 2 diabetes influences intraepithelial corneal nerve parameters and corneal stromal-epithelial nerve penetration sites. J. Diabetes Investig..

[47] Southam K., de Sousa C., Daniel A., Taylor B.V., Foa L., Premilovac D. (2022). Development and characterisation of a rat model that exhibits both metabolic dysfunction and neurodegeneration seen in type 2 diabetes. J. Physiol..

[48] Tavakoli M., Quattrini C., Abbott C., Kallinikos P., Marshall A., Finnigan J., Morgan P., Efron N., Boulton A.J., Malik R.A. (2010). Corneal confocal microscopy: A novel noninvasive test to diagnose and stratify the severity of human diabetic neuropathy. Diabetes Care.

[49] Davidson E.P., Coppey L.J., Yorek M.A. (2012). Early loss of innervation of cornea epithelium in streptozotocin-induced type 1 diabetic rats: Improvement with ilepatril treatment. Investig. Ophthalmol. Vis. Sci..

[50] Yu F.X., Lee P.S.Y., Yang L., Gao N., Zhang Y., Ljubimov A.V., Yang E., Zhou Q., Xie L. (2022). The impact of sensory neuropathy and inflammation on epithelial wound healing in diabetic corneas. Prog. Retin. Eye Res..

[51] Jia X., Wang X., Wang X., Pan Q., Xian T., Yu X., Guo L. (2018). In Vivo Corneal Confocal Microscopy Detects Improvement of Corneal Nerve Parameters following Glycemic Control in Patients with Type 2 Diabetes. J. Diabetes Res..

[52] Han S.B., Yang H.K., Hyon J.Y. (2019). Influence of diabetes mellitus on anterior segment of the eye. Clin. Interv. Aging.

[53] Sandireddy R., Yerra V.G., Areti A., Komirishetty P., Kumar A. (2014). Neuroinflammation and oxidative stress in diabetic neuropathy: Futuristic strategies based on these targets. Int. J. Endocrinol..

[54] Román-Pintos L.M., Villegas-Rivera G., Rodríguez-Carrizalez A.D., Miranda-Díaz A.G., Cardona-Muñoz E.G. (2016). Diabetic Polyneuropathy in Type 2 Diabetes Mellitus: Inflammation, Oxidative Stress, and Mitochondrial Function. J. Diabetes Res..

[55] Yang C., Zhao X., An X., Zhang Y., Sun W., Zhang Y., Duan Y., Kang X., Sun Y., Jiang L. (2023). Axonal transport deficits in the pathogenesis of diabetic peripheral neuropathy. Front. Endocrinol..

[56] Davidson E.P., Coppey L.J., Kardon R.H., Yorek M.A. (2014). Differences and similarities in development of corneal nerve damage and peripheral neuropathy and in diet-induced obesity and type 2 diabetic rats. Investig. Ophthalmol. Vis. Sci..

[57] Ferdousi M., Azmi S., Petropoulos I.N., Fadavi H., Ponirakis G., Marshall A., Tavakoli M., Malik I., Mansoor W., Malik R.A. (2015). Corneal Confocal Microscopy Detects Small Fibre Neuropathy in Patients with Upper Gastrointestinal Cancer and Nerve Regeneration in Chemotherapy Induced Peripheral Neuropathy. PLoS ONE.

[58] Zhang Y., Fan D., Zhang Y., Zhang S., Wang H., Liu Z., Wang H. (2021). Using corneal confocal microscopy to compare Mecobalamin intramuscular injections vs oral tablets in treating diabetic peripheral neuropathy: A RCT. Sci. Rep..

[59] Davidson E.P., Coppey L.J., Shevalye H., Obrosov A., Kardon R.H., Yorek M.A. (2017). Impaired corneal sensation and nerve loss in a type 2 rat model of chronic diabetes is reversible with combination therapy of menhaden oil, α-lipoic acid and enalapril. Cornea.

[60] Tavakoli M., Kallinikos P., Iqbal A., Herbert A., Fadavi H., Efron N., Boulton A.J., Malik R.A. (2011). Corneal confocal microscopy detects improvement in corneal nerve morphology with an improvement in risk factors for diabetic neuropathy. Diabet. Med..

[61] Kallinikos P., Berhanu M., O’Donnell C., Boulton A.J., Efron N., Malik R.A. (2004). Corneal nerve tortuosity in diabetic patients with neuropathy. Investig. Ophthalmol. Vis. Sci..

[62] Klisser J., Tummanapalli S.S., Kim J., Chiang J.C.B., Khou V., Issar T., Naduvilath T., Poynten A.M., Markoulli M., Krishnan A.V. (2022). Automated analysis of corneal nerve tortuosity in diabetes: Implications for neuropathy detection. Clin. Exp. Optom..

[63] Feldman E.L., Callaghan B.C., Pop-Busui R., Zochodne D.W., Wright D.E., Bennett D.L., Bril V., Russell J.W., Viswanathan V. (2019). Diabetic neuropathy. Nat. Rev. Dis. Primers.

[64] Pritchard N., Edwards K., Dehghani C., Fadavi H., Jeziorska M., Marshall A., Petropoulos I.N., Ponirakis G., Russell A.W., Sampson G.P. (2014). Longitudinal assessment of neuropathy in type 1 diabetes using novel ophthalmic markers (LANDMark): Study design and baseline characteristics. Diabetes Res. Clin. Pract..

[65] Davidson E.P., Holmes A., Coppey L.J., Yorek M.A. (2015). Effect of combination therapy consisting of enalapril, α-lipoic acid, and menhaden oil on diabetic neuropathy in a high fat/low dose streptozotocin treated rat. Eur. J. Pharmacol..

[66] Gad H., Mohammed I., Dauleh H., Pasha M., Al-Barazenji T., Hussain K., Malik R.A. (2024). Case report: Nerve fiber regeneration in children with melanocortin 4 receptor gene mutation related obesity treated with semaglutide. Front. Endocrinol..

[67] Mehta B.K., Nerkar D., Banerjee S. (2017). Characterization of peripheral neuropathy in rat model of type 2 diabetes. Indian J. Pharm. Educ. Res..

[68] Alharthy K.M., Balaha M.F., Devi S., Altharawi A., Yusufoglu H.S., Aldossari R.M., Alam A., di Giacomo V. (2023). Ameliorative Effects of Isoeugenol and Eugenol against Impaired Nerve Function and Inflammatory and Oxidative Mediators in Diabetic Neuropathic Rats. Biomedicines.

[69] Yamaguchi M., Noda-Asano S., Inoue R., Himeno T., Motegi M., Hayami T., Nakai-Shimoda H., Kono A., Sasajima S., Miura-Yura E. (2024). Dipeptidyl Peptidase (DPP)-4 Inhibitors and Pituitary Adenylate Cyclase-Activating Polypeptide, a DPP-4 Substrate, Extend Neurite Outgrowth of Mouse Dorsal Root Ganglia Neurons: A Promising Approach in Diabetic Polyneuropathy Treatment. Int. J. Mol. Sci..

[70] Panou T., Gouveri E., Popovic D.S., Papazoglou D., Papanas N. (2025). The Therapeutic Potential of Dipeptidyl Peptidase 4 Inhibitors and Glucagon-Like Peptide-1 Receptor Agonists in Diabetic Peripheral Neuropathy. Diabetes Ther..

[71] Williams-Herman D., Engel S.S., Round E., Johnson J., Golm G.T., Guo H., Musser B.J., Davies M.J., Kaufman K.D., Goldstein B.J. (2010). Safety and tolerability of sitagliptin in clinical studies: A pooled analysis of data from 10,246 patients with type 2 diabetes. BMC Endocr. Disord..

[72] Huai Y., Dong Y., Xu J., Meng N., Song C., Li W., Lv P. (2013). L-3-n-butylphthalide protects against vascular dementia via activation of the Akt kinase pathway. Neural Regen. Res..

[73] Liu Y., You M., Wang J., Liu J., Zhou X., Kenny J., Rong R., Xia X. (2025). 3-n-Butylphthalide exerts mitochondria-mediated retinal ganglion cell protection and modulates eye-brain axis-related emotional disturbances in glaucoma. Int. J. Surg..

[74] Shayani Rad M., Moohebati M., MohammadEbrahimi S., Motamedshariaty V.S., Mohajeri S.A. (2022). Safety evaluation and biochemical efficacy of celery seed extract (*Apium graveolens*) capsules in hypertensive patients: A randomized, triple-blind, placebo-controlled, cross-over, clinical trial. Inflammopharmacology.

[75] Escobedo-Gutiérrez M.J., Cortez-Navarrete M., Martínez-Abundis E., Pérez-Rubio K.G. (2026). Effect of Celery Seed (*Apium graveolens* L.) Administration on the Components of Metabolic Syndrome, Insulin Sensitivity, and Insulin Secretion: A Clinical Trial. Pharmaceuticals.

[76] Gao H., Lv G.-h., Huang R.-w. (2025). A Narrative Pharmacological Review: Current Research Status of in vivo and in vitro Applications of Natural Spice Celery Seed. Nat. Prod. Commun..

[77] Petropoulos I.N., Ferdousi M., Marshall A., Alam U., Ponirakis G., Azmi S., Fadavi H., Efron N., Tavakoli M., Malik R.A. (2015). The Inferior Whorl For Detecting Diabetic Peripheral Neuropathy Using Corneal Confocal Microscopy. Investig. Ophthalmol. Vis. Sci..

[78] Ferdousi M., Kalteniece A., Petropoulos I., Azmi S., Dhage S., Marshall A., Boulton A.J.M., Efron N., Faber C.G., Lauria G. (2020). Diabetic Neuropathy Is Characterized by Progressive Corneal Nerve Fiber Loss in the Central and Inferior Whorl Regions. Investig. Ophthalmol. Vis. Sci..

[79] Lu K.Y., Lin S.Z., Primus Dass K.T., Lin W.J., Liu S.P., Harn H.J. (2021). 3-N-butylphthalide protects against high-fat-diet-induced obesity in C57BL/6 mice and increases metabolism in lipid-accumulating cells. Biomed. Pharmacother..

