# Supplementary material for: Effects of Sitagliptin and Celery Seed Extract on Corneal Nerve Morphology and Sensory Dysfunction in Diabetic Rats
Source: Nutrients. 2026 Jul 9;18(14):2243. doi: 10.3390/nu18142243 (PMC13415245; doi:10.3390/nu18142243)

Supplementary Materials

LC–MS Characterisation of Celery Seed Extract

Supplementary Figure S1

Representative PDA (254 nm) and LC–MS total ion chromatograms of the celery seed extract used in this study. The PDA chromatogram revealed a major peak at a retention time of 6.018 min together with several minor peaks. Corresponding signals were observed in the LC–MS total ion chromatogram, demonstrating the presence of multiple detectable constituents within the extract. The predominant chromatographic peak observed at approximately 6.0 min was selected for subsequent mass spectral analysis

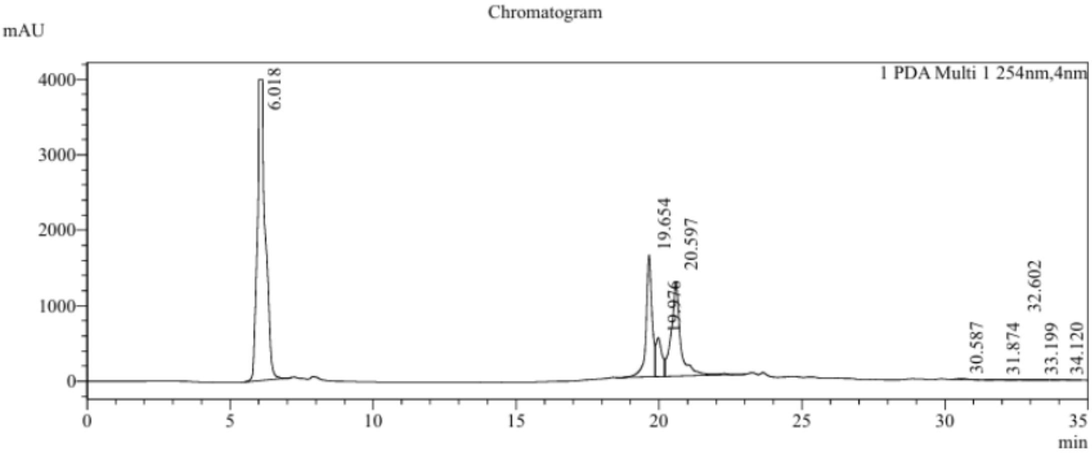

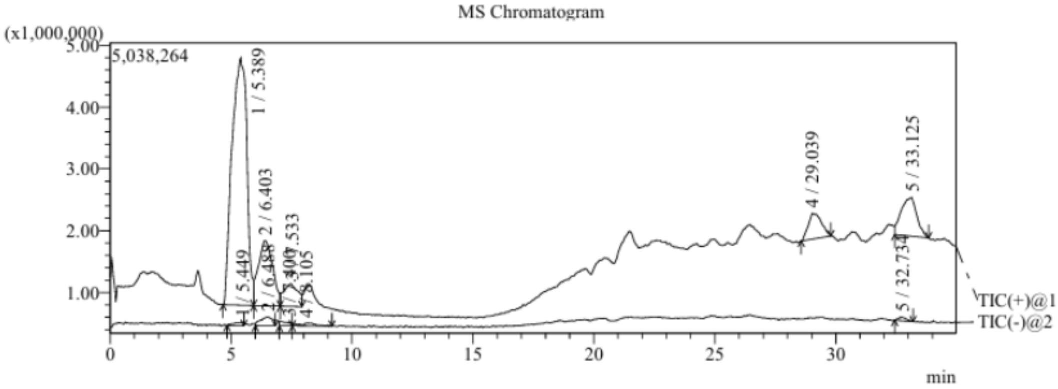

Supplement: Supplementary file 1 [file nutrients-18-02243-s001.zip › nutrients-4366164-supplementary.pdf]
